# Effect of coronavirus disease 2019 on recurrences and follow up of head and neck squamous cell carcinoma

**DOI:** 10.1017/S0022215121000918

**Published:** 2021-03-23

**Authors:** E Kytö, E Haapio, I Kinnunen, H Irjala

**Affiliations:** Department of Otorhinolaryngology – Head and Neck Surgery, Turku University Hospital and University of Turku, Turku, Finland

**Keywords:** Head And Neck Cancer, Follow-Up Care, COVID-19, Recurrence, Telemedicine

## Abstract

**Objective:**

This prospective study aimed to evaluate possible diagnostic delays in head and neck squamous cell carcinoma recurrences due to the changed follow-up protocol during the coronavirus disease 2019 pandemic.

**Methods:**

The follow-up appointments of head and neck squamous cell carcinoma patients treated more than one year prior to the pandemic were changed to telephone appointments in order to reduce physical visits to the hospital. All contacts, reasons for contact and recurrent cancers were recorded.

**Results:**

There were 17 recurrences during a seven-month study period among 178 patients treated in the previous year (10 per cent); 14 of these recurrences occurred in patients whose treatment had ended less than one year previously and 3 occurred more than one year after treatment had ended. There was no delay in diagnoses of recurrent tumours or treatment despite reduced visits because of the coronavirus disease 2019 pandemic.

**Conclusion:**

According to our analyses, no delay was caused in the diagnoses of recurrent diseases. Follow up by telephone or telemedicine can be considered as part of the follow-up protocol one year after the treatment of head and neck squamous cell carcinoma when necessary.

## Introduction

Head and neck squamous cell carcinoma (SCC) is the sixth most common malignancy globally. Its incidence is rising, especially in the oral cavity and oropharynx, and patients are developing this cancer at a younger age.^1^ More than 50 per cent of head and neck SCC patients develop regional or distant relapses, and even stage I diseases recur. Thus, the follow up of head and neck cancer patients is important.^2^ The majority of recurrent tumours are found within one year of the end of treatment.^3^

Coronavirus disease 2019 (Covid-19) was identified in Wuhan, China, in early 2020, and is caused by severe acute respiratory syndrome coronavirus-2 (SARS-CoV-2). The worldwide pandemic changed the follow-up protocols and guidelines for head and neck cancer patients in March 2020. The need to reduce in-person out-patient visits was evident.

At first, in this new era, guidelines for how to manage the treatment and follow up of head and neck cancers were lacking. The recommendations for follow up after treatment were to reduce all follow-up visits and use telemedicine.^4–6^ Singh *et al*. published a review article on using telemedicine applications in otorhinolaryngology, and recommended telemedicine follow up for head and neck cancer.^7^

In light of the Covid-19 pandemic, we modified the follow-up protocol in order to reduce the number of patients visiting our tertiary care academic centre (the Department of Otorhinolaryngology – Head and Neck Surgery, Turku University Hospital, Finland), and subsequently investigated the effects of the changed protocol. This prospective study aimed to evaluate possible diagnostic delays in head and neck SCC recurrences due to the changed follow-up protocol during the Covid-19 pandemic.

## Materials and methods

This prospective study covered all cases of head and neck SCC. The routine follow-up clinic appointments of head and neck SCC patients treated more than one year previously at Turku University Hospital were systematically changed to telephone appointments from 23 March to 27 May 2020. A registered nurse contacted all the patients whose treatment had ended more than one year prior and informed them of the new practice. Only one routine follow-up visit was substituted by a telephone appointment for each of these patients. Their subsequent visit was normal and included a full physical examination. Follow-up visits remained unchanged for patients whose treatment had ended less than one year previously. Some of these patients refused to come to the appointment because of the pandemic situation and their appointment was conducted by telephone. The data of these patients were collected until 23 October 2020.

During the seven months (March to October 2020), all patient contacts, reasons for contact and detected recurrent cancers were carefully recorded. We studied any possible associations between delayed diagnosis and the Covid-19 pandemic. This study was approved by the Institutional Research Ethics Board of Turku University Hospital (record number: T248/2020).

## Results

**Fig. 1.**
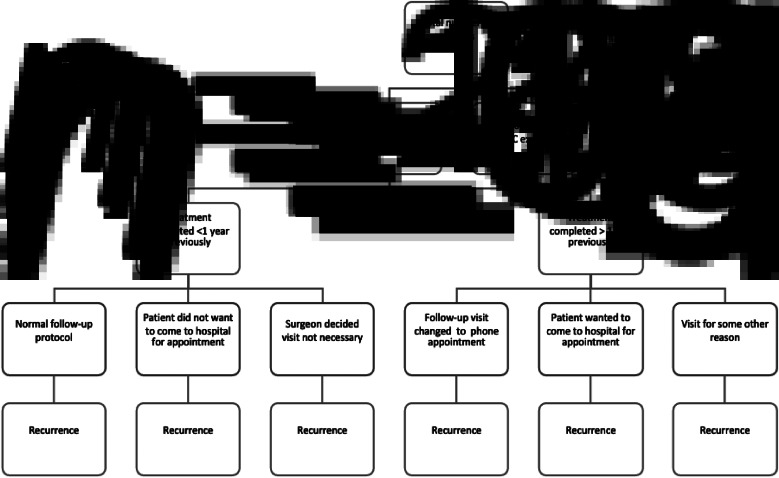
Flowchart of patient follow up during coronavirus disease 2019 pandemic. HNSCC = head and neck squamous cell carcinoma

The modified follow-up protocol was used from 23 March to 27 May 2020. A total of 209 patients with head and neck malignancy were followed up during this period. Those with histology findings other than squamous cellular carcinoma were excluded, leaving 178 patients. Head and neck SCC patients were divided into two main groups, and these two groups were further divided into three subgroups according to time since treatment and type of follow up (Figure 1).

After the lockdown period, 169 of the 178 head and neck SCC patients (95 per cent) visited the out-patient clinic before 23 October 2020. This included 87 of the 96 patients (91 per cent) whose follow up was conducted only by telephone during the first Covid-19 spring period (23 March to 27 May 2020).

During the seven-month study period, 17 head and neck SCC patients (10 per cent) were diagnosed with a recurrent cancer. Fourteen (82 per cent) of these recurrent tumours were found in patients whose treatment had ended less than one year previously. These patients underwent the normal control protocol during the spring period, except for one patient who did not want to come to the out-patient clinic because of the pandemic. In three patients (18 per cent), recurrence occurred more than one year after their treatment had ended.

In addition to the scheduled follow-up visits, 39 of the 178 patients consulted the hospital because of their symptoms during this study period. Twenty-six of these patients were concerned with symptoms directly related to possible recurrence, whereas 13 contacted the hospital for some other reason. Nine of these 39 patients who contacted the hospital suffered recurrent disease. For eight of these nine patients, treatment had ended less than one year previously.

We found six other cases of recurrence among patients whose treatment had ended less than one year previously. Three of these were found during a routine follow-up visit by physical examination. All these patients also had symptoms. One of these three patients was the individual who did not want to visit the out-patient clinic during the lockdown period. Recurrence was not observed in this patient at the next visit after the telephone appointment, but was found later during the second visit after the telephone appointment, by which time the patient was symptomatic. The other three patients with recurrence had distant metastases. Two of the distant metastases were found on baseline positron emission tomography magnetic resonance imaging (PET-MRI) three months after treatment. Neither of these patients had any symptoms. The third case of distant metastases was also found on PET-MRI, a year after treatment, and the patient had no symptoms.

In the group whose treatment had ended more than one year previously, three patients wanted to visit the out-patient clinic instead of the offered telephone appointment, but none of these had recurrent disease. Eight patients were invited to an appointment following the surgeon's request after the telephone call appointment, but no recurrences were found among these patients.

We found three recurrences among the patients whose treatment had ended more than one year previously. The first patient attended a follow-up visit for sinonasal SCC at the clinic instead of a telephone appointment. This was because metastasis had been detected on routine PET-MRI, carried out when the patient had no symptoms, before the scheduled visit. The second patient was treated for SCC of the vocal folds and had no symptoms to report on the telephone; however, one month later, when the patient was examined because of fatigue, we found a second primary carcinoma in their lung. The third patient had previously had tonsillar cancer and, because of their symptoms, an additional visit was arranged, during which a second primary SCC of the nasal cavity was detected. Analysis of these three cases revealed that none of their diagnoses were delayed because of Covid-19.

## Discussion

At the beginning of the Covid-19 pandemic, the use of telemedicine was recommended instead of follow-up visits.^7^ Otolaryngologists were at a high risk because of aerosol-generating procedures, as in the SARS-CoV-1 pandemic in 2003.^8^ Indeed, the first reported physician fatality in the Covid-19 pandemic was an otolaryngologist in Wuhan, China.^9^ The first published guidelines recommended protecting physicians from infection when treating head and neck cancers, and minimising the number of all surgical and mucosal aerosol-generating procedures conducted.^10–12^

The follow up of head and neck cancer patients is controversial. Three major head and neck societies (American Society for Head and Neck Surgery, Society of Head and Neck Surgery, and British Association of Head and Neck Oncologists) recommend a follow-up protocol of at least five years.^13,14^ Many studies have challenged this five-year protocol.^15–17^ In our own follow-up study, we found that routine head and neck SCC follow-up visits can be reduced after 3 years, as all recurrences after 34 months were found as a result of new symptoms.^3^ In Finland, the follow-up protocol ends three years after treatment completion. However, even after three years, patients can contact the clinic in the event of symptoms and an appointment will be arranged with the head and neck surgeon.

The high number of symptomatic patients among the recurrences in this prospective study is noteworthy. Twelve of the 17 patients with recurrence (71 per cent) had symptoms, and 9 of these actively contacted the hospital because of symptoms. This is significantly more than in our earlier retrospective study, in which only 56 per cent of the recurrent cases reported symptoms.^3^ This may be because cases were recorded in more detail in a prospective setting, or because patient information was more successful; after the first study, we have tried even harder to actively encourage patients to contact the hospital immediately if any new symptoms occur.

Patients whose treatment had ended less than one year previously attended follow-up appointments as normal, with the exception of three patients who requested a telephone appointment instead of a visit, and six patients for whom the surgeon recommended a telephone appointment instead of a visit. Fourteen recurrences were found in this group of patients whose treatment had ended less than one year previously. None of these diagnoses were delayed because of the changed protocol due to the Covid-19 pandemic. Thirteen of these 14 patients attended normal follow-up routine clinical examinations, and 1 requested a telephone appointment instead.

In the group of patients whose treatment had ended over a year earlier, only three recurrent tumours were found. Two of these were in the lungs – one was metastasis and one was a second primary; both cases were revealed by radiological imaging. The third recurrence was a second primary in the nasal cavity; in this case, the patient contacted the hospital because of symptoms. As in these first two cases, usually no findings are detected in a normal physical examination if the recurrence is in the lungs, which is where the distant metastases are often located in head and neck SCC. Patients are able to describe symptoms on the telephone, for example coughing, haemoptysis, weight loss and dyspnoea; hence, in this sense, a telephone appointment is comparable to hospital appointments in the follow-up period after one year of treatment for head and neck SCC. In our study, the routine positron emission tomography scan was also effective for detecting recurrences.

• The follow-up protocol of head and neck squamous cell carcinoma patients was changed as a result of the coronavirus disease 2019 pandemic
• There were 17 recurrences during a seven-month period among 178 patients
• This study detected no delay in the diagnoses of recurrent diseases
• Some out-patient clinic visits may be replaced by a telephone call

The results of this study show that no delay in diagnoses or unexpected recurrences occurred because of the Covid-19 pandemic among patients with previously treated head and neck SCC. Despite the short time period and small population of this study, our findings encourage further evaluation of the rationale for the normal follow-up protocol, especially when more than one year has passed since the end of treatment. It is suggested that some out-patient clinic visits could be replaced by a telephone call. Follow up by telephone or telemedicine might be less stressful for patients who are nervous about visiting the hospital. It could also be cost-effective. Further studies are needed to investigate the potential of telephone appointments and telemedicine for the follow up of head and neck SCC patients.

## Conclusion

In light of the Covid-19 pandemic, the follow-up protocol of head and neck SCC patients treated more than one year earlier was changed in order to reduce the number of physical visits to hospitals. Patients were contacted by telephone and invited to out-patient clinic appointments only if needed. This study detected no delay in the diagnoses of recurrent diseases. This encourages critical evaluation of the follow-up protocol in non-pandemic circumstances also.

## References

[1] Chaturvedi AK, Anderson WF, Lortet-Tieulent J, Curado MP, Ferlay J, Franceschi S Worldwide trends in incidence rates for oral cavity and oropharyngeal cancers. J Clin Oncol 2013;31:4550–9 24248688 10.1200/JCO.2013.50.3870 PMC3865341

[2] Argiris A, Karamouzis MV, Raben D, Ferris RL. Head and neck cancer. Lancet 2008;371:1695–709 18486742 10.1016/S0140-6736(08)60728-X PMC7720415

[3] Kytö E, Haapio E, Minn H, Irjala H. Critical review of the follow-up protocol for head and neck cancer patients. J Laryngol Otol 2019;133:424–9 31006389 10.1017/S0022215119000811

[4] BAHNO Statement on COVID-19: Initial guidance for head and neck cancer management during Covid-19 pandemic in consultation with ENT UK. BAOMS endorsement awaited. In: https://bahno.org.uk/bahno_statement_on_covid-19.aspx [17 March 2020]

[5] Han AY, Miller JE, Long JL, St John MA. Time for a paradigm shift in head and neck cancer management during the COVID-19 pandemic. Otolaryngol Head Neck Surg 2020;163:447–54 32484380 10.1177/0194599820931789 PMC7484111

[6] Kanatas A, Rogers SN. The role of the Head and Neck cancer-specific Patient Concerns Inventory (PCI-HN) in telephone consultations during the COVID-19 pandemic. Br J Oral Maxillofac Surg 2020;58:497–9 32340765 10.1016/j.bjoms.2020.04.010 PMC7165278

[7] Singh AK, Kasle DA, Jiang R, Sukys J, Savoca EL, Lerner M A review of telemedicine applications in otorhinolaryngology: considerations during the coronavirus disease of 2019 pandemic. Laryngoscope 2021;131:744–59 32942340 10.1002/lary.29131 PMC7537247

[8] Drosten C, Günther S, Preiser W, van der Werf S, Brodt HR, Becker S Identification of a novel coronavirus in patients with severe acute respiratory syndrome. N Engl J Med 2003;348:1967–76 12690091 10.1056/NEJMoa030747

[9] Chan JYK, Wong EWY, Lam W. Practical aspects of otolaryngologic clinical services during the 2019 novel coronavirus epidemic: an experience in Hong Kong. JAMA Otolaryngol Head Neck Surg 2020;146:519–20 32196070 10.1001/jamaoto.2020.0488

[10] Kowalski LP, Sanabria A, Ridge JA, Ng WT, Bree R, Rinaldo A COVID-19 pandemic: effects and evidence-based recommendations for otolaryngology and head and neck surgery practice. Head Neck 2020;42:1259–67 32270581 10.1002/hed.26164 PMC7262203

[11] American Academy of Otolaryngology – Head and Neck Surgery. Tracheotomy recommendations during the COVID-19 pandemic. In: https://entnet.org/content/tracheotomy-recommendations-during-covid-19-pandemic [2 April 2020]

[12] American Head and Neck Society. How COVID-19 is Affecting our Head and Neck Community. In: www.ahns.info/wp-content/uploads/2020/03/AHNS-Statement.pdf [23 March 2020]

[13] Paniello RC, Virgo KS, Johnson MH, Clemente MF, Johnson FE. Practice patterns and clinical guidelines for posttreatment follow-up of head and neck cancers: a comparison of 2 professional societies. Arch Otolaryngol Head Neck Surg 1999;125:309–13 10190803 10.1001/archotol.125.3.309

[14] Simo R, Homer J, Clarke P, Mackenzie K, Paleri V, Pracy P Follow-up after treatment for head and neck cancer: United Kingdom National Multidisciplinary Guidelines. J Laryngol Otol 2016;130:S208–11 27841136 10.1017/S0022215116000645 PMC4873918

[15] Ritoe SC, Krabbe PFM, Kaanders JHAM, Van Den Hoogen FJA, Verbeek ALM, Marres HAM. Value of routine follow-up for patients cured of laryngeal carcinoma. Cancer 2004;101:1382–9 15368326 10.1002/cncr.20536

[16] Liu G, Dierks EJ, Bell RB, Bui TG, Potter BE. Post-therapeutic surveillance schedule for oral cancer: is there agreement? Oral Maxillofac Surg 2012;16:327–40 22941063 10.1007/s10006-012-0356-3

[17] Cancer Care Ontario. The Management of Head and Neck Cancer in Ontario. In: https://www.cancercareontario.ca/en/guidelines-advice/types-of-cancer/536 [15 December 2009]

